# Integrated analysis of the roles and prognostic value of RNA binding proteins in lung adenocarcinoma

**DOI:** 10.7717/peerj.8509

**Published:** 2020-02-06

**Authors:** Wei Li, Na Li, Lina Gao, Chongge You

**Affiliations:** 1 Laboratory Medicine Center, Lanzhou University Second Hospital, Langzhou, China; 2 Department of Pathology, the First Affiliated Hospital of Hunan University of Medicine, Huaihua, China

**Keywords:** Lung adenocarcinoma, RNA binding proteins, Post-transcriptional regulation, Prognostic value, Integrated bioinformatics analysis

## Abstract

Lung cancer is the top cause of carcinoma-associated deaths worldwide. RNA binding proteins (RBPs) dysregulation has been reported in various malignant tumors, and that dysregulation is closely associated with tumorigenesis and tumor progression. However, little is known about the roles of RBPs in lung adenocarcinoma (LUAD). In this study, we downloaded the RNA sequencing data of LUAD from The Cancer Genome Atlas (TCGA) database and determined the differently expressed RBPs between normal and cancer tissues. We then performed an integrative analysis to explore the expression and prognostic significance of these RBPs. A total of 164 differently expressed RBPs were identified, including 40 down-regulated and 124 up-regulated RBPs. Pathway and Gene ontology (GO) analysis indicated that the differently expressed RBPs were mainly related to RNA processing, RNA metabolic process, RNA degradation, RNA transport, splicing, localization, regulation of translation, RNA binding, TGF-beta signaling pathway, mRNA surveillance pathway, and aminoacyl-tRNA biosynthesis. Survival analysis revealed that the high expression of *BOP1* or *GNL3* or *WDR12* or *DCAF13* or *IGF2BP3* or *IGF2BP1* were associated with poor overall survival (OS). Conversely, overexpression of *KHDRBS2*/*SMAD* predicted high OS in these patients. ROC curve analysis showed that the eight hub genes with a better diagnostic accuracy to distinguish lung adenocarcinoma. The results provided novel insights into the pathogenesis of LUAD and the development of treatment targets and prognostic molecular markers.

## Introduction

RNA-binding proteins (RBPs) are generally recognized as proteins that bind to a variety of RNAs, such as rRNAs, miRNAs, snRNAs, ncRNAs, mRNAs, snoRNAs, and tRNAs. Currently, there are more than 1,500 experimentally validated RBP coding genes in human genome, accounting for about 7.5% of all protein-coding genes (Gerstberger, Hafner & Tuschl, 2014). They can interact with other proteins or RNAs to form ribonucleoprotein complexes that regulate mRNA stability, RNA localization, export, processing, splicing, degradation, and translation (Masuda & Kuwano, 2019). The RBP decides the function and durability of each transcript and maintains cellular homeostasis (Masuda & Kuwano, 2019). Since the RBPs play pivotal roles in post-transcriptional gene expression, it is not surprising that differentially expressed RBPs are closely associated with the pathogenesis and progression of various diseases. Mutations in RBP *LIN28A* cause embryonic stem cell developmental defects and a Parkinson’s disease-related phenotype (Chang et al., 2019). ZFP36L1 is an RBP protect against Osteoarthritis pathogenesis by regulating the HSP70 family members to inhibit chondrocyte apoptosis (Son et al., 2019). Besides, previous studies have shown that the RBPs such as SRSF1, HuR, Rbm38, and QKI, as critical post-transcriptional regulators, play essential roles in the occurrence and development of cardiovascular diseases (De Bruin et al., 2017; Sonnenschein et al., 2019). Although RBPs be closely related to the initiation and progression of human diseases, the general roles of RBPs in tumor are still unclear.

To date, more and more studies have revealed that RBPs were dysregulated in tumors, which influence RNA modification and protein translation, involved in cancer development and progression (Legrand, Dixon & Sobolewski, 2019; Wang et al., 2020; Chatterji & Rustgi, 2018). For instance, RBP CELF6 served as a potential tumor suppressor. It is transcriptional silencing in human breast cancer due to hypermethylation modification of its promoter (Pique et al., 2019); RBP SORBS2 inhibit liver cancer carcinogenesis and metastasis by regulating the stability of RORA (Han, Huang & Zhang, 2019); AGO2 promote tumor development by up-regulating the expression of oncogenic miR-19b (Zhang et al., 2019); RBP KHSRP promotes lung cancer cell proliferation, migration, and invasion (Yan et al., 2019); HuR by regulating related mRNA stability to induce tumor cell proliferation and metastasis of stomach carcinoma (Xie et al., 2019b). However, the functions of most RBPs have not yet been determined in cancers. A systematic functional analysis of RBPs will help us thoroughly investigate its role in tumors.

Lung cancer is a very harmful disease with an average 5-year relative survival rate of only 18% (Siegel, Miller & Jemal, 2018). In recent years, numerous diagnostic molecular markers related to lung tumor have been identified (Xie et al., 2019a; Bao et al., 2017; Tepeli et al., 2012; Jan et al., 2019; Sun et al., 2019), but it is difficult to achieve accurate early-stage detection. This is probably the most important cause of high mortality in lung cancer patients. Therefore, there is a pressing need to develop an effective means for early detection and diagnosis to improve the treatment of lung cancer. The large-scale tumor genome project provides a wealth of gene expression data, which gives us an excellent opportunity to identify potential tumor molecular markers. Herein, we obtained LUAD RNA-sequencing data with corresponding clinical information from the cancer genome atlas (TCGA) database. Then, a series of bioinformatics methods were used to identify the differential expression of RPBs in tumor and normal tissues and to analyze the potential functional and clinical significance of these RBPs. Our results have displayed a number of RBPs associated with the pathogenesis of LUAD, which might be potentially helpful for developing diagnosis and prognosis biomarkers.

## Material and Methods

### Data acquisition and processing

The RNA-sequencing information of 524 LUAD samples and 59 normal lung tissue samples with corresponding clinical information were obtained from TCGA database (https://portal.gdc.cancer.gov/). The DESeq2 package (http://www.bioconductor.org/packages/release/bioc/html/DESeq2. html) was used to preprocessed raw data and excluded genes with an average count value less than 1. Besides, we also used DESeq2 package to identify the differently expressed RBPs in view of —log_2_ fold change (FC)— ≥1 and false discovery rate (FDR)<0.05. The GSE30219 dataset were downloaded from Gene Expression Omnibus (GEO) (https://www.ncbi.nlm.nih.gov/geo/) and used as a validation cohort.

### GO and pathway enrichment analysis

In order to explore the function of the differently expressed RBPs, GO enrichment and Kyoto Encyclopedia of Genes and Genomes (KEGG) pathway analysis were performed using WEB-based Gene Set Analysis Toolkit (WebGestalt, http://www.webgestalt.org/) (Liao et al., 2019). Both *P* and FDR values were less than 0.05 were considered statistically significant.

### Protein–protein interaction (PPI) network construction and module selecting

The PPI network is an effective means for exploring the association between proteins in various organisms. In this study, the STRING database (http://www.string-db.org/) (Szklarczyk et al., 2019) were used to identify protein-protein interaction information of the differently expressed RBPs. The PPI pairs identified by STRING database were imported into the Cytoscape 3.7.0 software to build a PPI network. The key modules and hub genes were identified by using Molecular Complex Detection (MCODE) plug-in with both MCODE score and node counts number more than 5. All *P* ≤** 0.05 were chosen as the significant threshold.

### Survival analysis

The prognostic value of key genes screed from important modules in PPI network were analyzed by using an online database, GEPIA (http://gepia.cancer-pku.cn/), which included gene expression data and corresponding survival information of 478 LUAD patients (Tang et al., 2017b). All LUAD patient samples were divided into low or high expression group according to median expression, then analyze the overall survival (OS) by GEPIA. Log-Rank test was used to assess the prognostic value and logrank *P* ≤ 0.05 was considered as a significant difference.

### Hub genes selection and efficacy evaluation

Given that the ten key genes from modules analysis were all up-regulated genes, we also run a survival analysis of the top 10 high expressed genes and top 20 low expressed genes, respectively. Taking together, the genes with *P* ≤** 0.05 in survival analysis were determined as real hub genes. Subsequently, the expression of these hub genes was verified at the translation and transcription levels by utilized the Human Protein Atlas (HPA) databases and GEPIA databases (http://www.proteinatlas.org/) (Uhlen et al., 2015). The receiver operating characteristic (ROC) curve was calculated with Graphpad prism 7.0 software to assess the capability to distinguish normal lung and LUAD tissue. Multivariate Cox regression analysis was performed on these hub genes to calculate a risk score of each patient. Subsequently, a RBPs-related prognostic signature based on the coefficient value of these hub genes was evaluated in TCGA and GSE30219 cohorts.

### Mutations and copy-number alterations analysis of key genes

The mutations and copy-number alterations information of all hub genes from were determined by utilizing segmentation analysis and GISTIC algorithm in cBioPortal (https://www.cbioportal.org/). Then, we conducted the co-expression analysis and constructed the network according to the cBioPortal’s instruction. In addition, functional enrichment analysis of hub genes alterations was evaluated by WebGestalt.

## Results

### Identifying the differently expressed RBPs in lung adenocarcinoma patients

**Figure 1:**
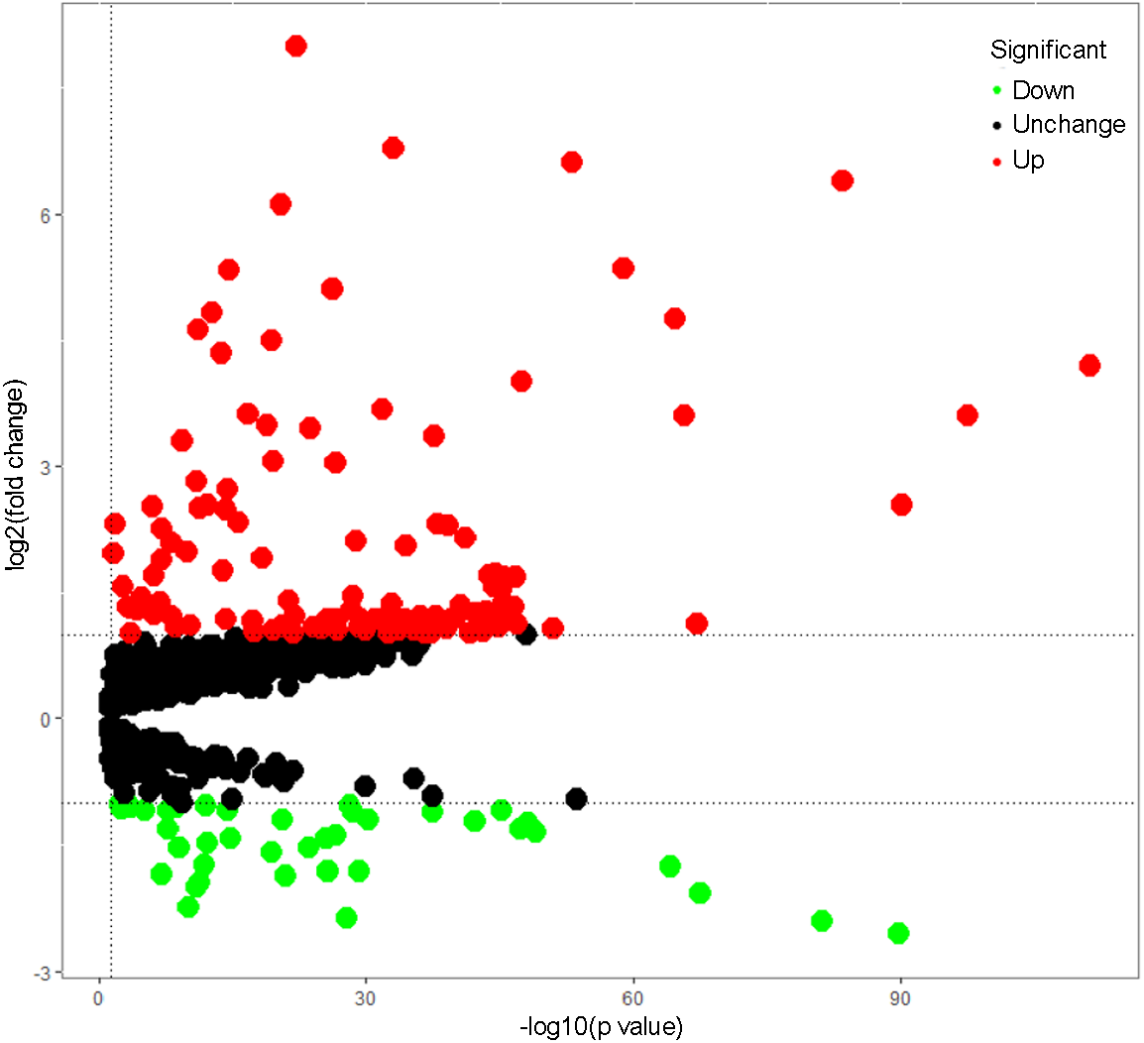
The differentially expressed RBPs in lung adenocarcinoma. Green: down-regulation with a fold change of more than 2; black: unchanged genes; red: up-regulation with fold change of more than 2.

Total 1542 RBPs (Gerstberger, Hafner & Tuschl, 2014) were included in this study, and 164 RBPs were identified as differentially expressed genes between tumors and normal samples (*P* < 0.05, —log_2_FC)— ≥1.0), including 40 down-regulated RBPs and 124 up-regulated RBPs (Table S1). The expression distribution of these differently expressed RBPs was showed in Fig. 1.

### Functional and pathway enrichment analysis of the differently expressed RBPs

We uploaded all differently expressed RBPs to the online tool WebGestalt for GO categories and KEGG pathways analysis. GO analysis results indicated that downregulated RBPs were significantly enriched in the biological processes (BP) related to mRNA processing, regulation of translation, regulation of cellular amide metabolic processes, and metabolic processes (Table 1). The upregulated RBPs were significantly enriched in biological processes, including amide biosynthetic processes, RNA processing, translation, peptide metabolic processes, peptide biosynthetic processes, and ncRNA metabolic processes (Table 1). For molecular function (MF), the downregulated RBPs were significantly enriched in RNA binding, single-stranded RNA binding, mRNA 3′-UTR AU-rich region binding and mRNA 3′-UTR binding (Table 1), while the upregulated RBPs were significantly enriched in RNA binding, catalytic activity acting on RNA, mRNA binding, and poly-pyrimidine tract binding (Table 1). The GO cellular component (CC) analysis showed that the decreased differently expressed RBPs were enriched in RNA cap binding complex, and ribonucleoprotein complex, and upregulated RBPs were mainly enriched in ribonucleoprotein complex, ribonucleoprotein granule, cytoplasmic ribonucleoprotein granule, nucleolus, and P granule (Table 1). In addition, results of KEGG pathway analysis indicated that downregulated RBPs were mainly enriched in TGF-beta signaling pathway, while upregulated RBPs were mainly enriched in pathways of mRNA surveillance pathway, RNA degradation, RNA transport, and aminoacyl-tRNA biosynthesis (Table 1).

**Table 1:** GO enrichment and KEGG pathway analysis of differently expressed RBPs.

	GO term	*P* value	FDR
Down-regulated RBPs			
	mRNA processing	3.68E−09	<0.0001
	mRNA metabolic process	2.43E−10	2.2102E−06
	RNA processing	1.86E−08	0.0001
	regulation of translation	2.60E−08	0.0001
	regulation of cellular amide metabolic process	9.26E−08	0.0002
	RNA cap binding complex	1.14E−06	0.0007
	ribonucleoprotein complex	1.19E−06	0.0007
	RNA binding	<0.0001	<0.0001
	mRNA binding	5.88E−11	5.52E−08
	single-stranded RNA binding	8.12E−10	5.08E−07
	mRNA 3′-UTR binding	1.65E−08	7.6E−06
	mRNA 3′-UTR AU-rich region binding	2.02E−08	7.6E−06
	TGF-beta signaling pathway	0.0004	0.0376
Up-regulated RBPs			
	RNA processing	<0.0001	<0.0001
	peptide metabolic process	<0.0001	<0.0001
	amide biosynthetic process	<0.0001	<0.0001
	peptide biosynthetic process	<0.0001	<0.0001
	translation	<0.0001	<0.0001
	ncRNA metabolic process	<0.0001	<0.0001
	ribonucleoprotein complex	<0.0001	<0.0001
	ribonucleoprotein granule	<0.0001	<0.0001
	cytoplasmic ribonucleoprotein granule	2.22E−16	8.70E−14
	nucleolus	8.46E−13	2.45E−10
	P granule	1.46E−12	2.45E−10
	RNA binding	<0.0001	<0.0001
	catalytic activity, acting on RNA	<0.0001	<0.0001
	mRNA binding	<0.0001	<0.0001
	poly-pyrimidine tract binding	5.45E−08	<0.0001
	translation regulator activity	1.39E−07	<0.0001
	mRNA surveillance pathway	<0.0001	<0.0001
	RNA degradation	<0.0001	<0.0001
	RNA transport	<0.0001	<0.0001
	Ribosome	0.0001	0.0001
	Aminoacyl-tRNA biosynthesis	0.0079	0.0079

### Protein-protein interaction network construction and key modules screening

**Figure 2:**
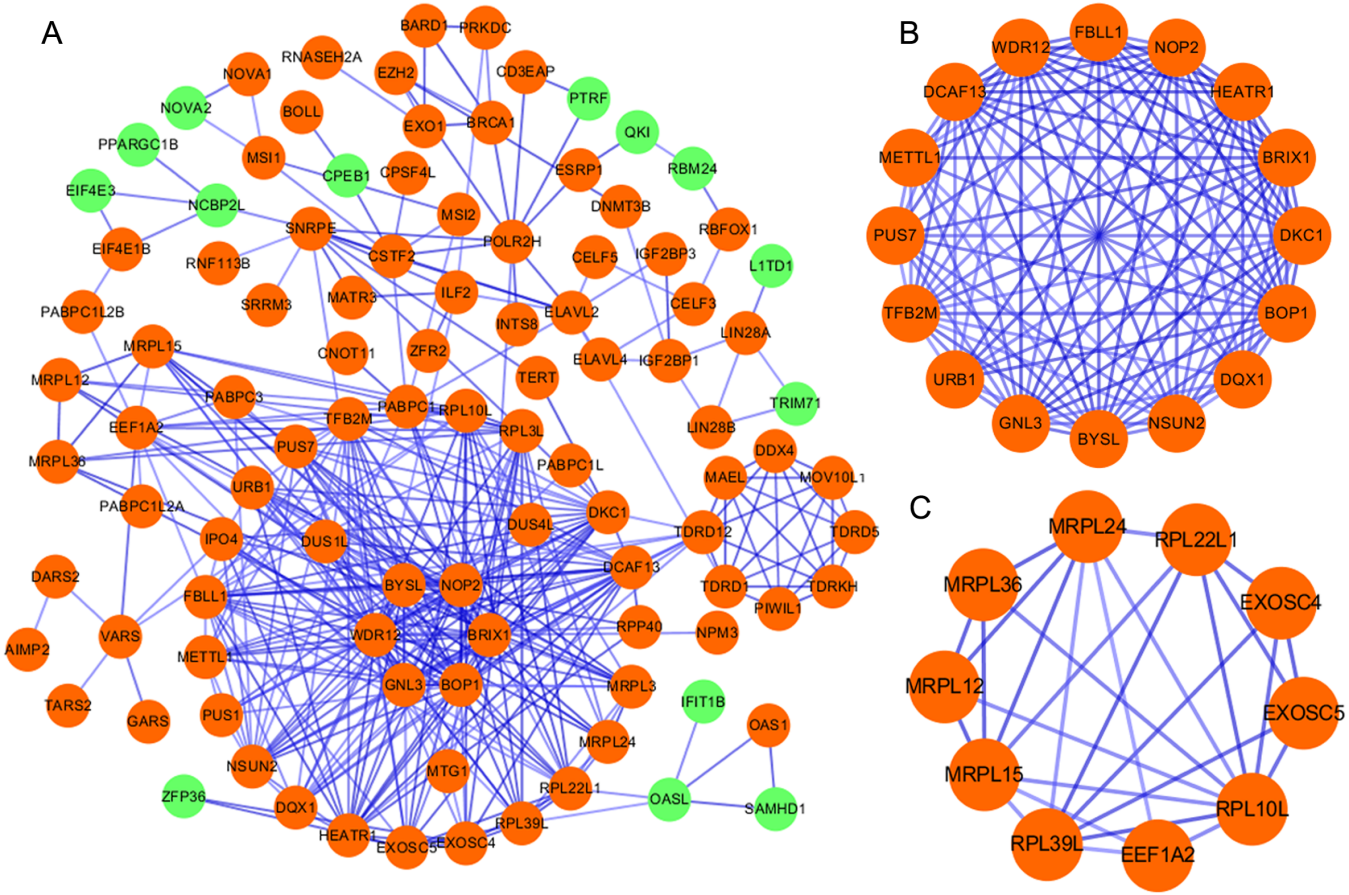
Protein–protein interaction network and modules analysis. (A) Protein–protein interaction network of RBPs; (B) critical module 1 from PPI network; (C) critical module 2 from PPI network. Green: down-regulation with a fold change of more than 2; orange: up-regulation with fold change of more than 2.

According to the information in the STRING database, we constructed a PPI network with 103 nodes and 360 edges by using Cytoscape software (Fig. 2A). Ten genes were screened as candidate hub genes by computing degree and betweenness, including *BOP1, GNL3, WDR12, NOP2, BYSL, BRIX1, DCAF13, TFB2M, NSUN2* and *DKC1.* Furthermore, the MODE plugin was used to identify possible key modules from the co-expression network and the top 2 significant modules were screened (Figs. 2B–2C). Function enrichment analysis revealed that the RBPs involved in module 1 were related to ncRNA processing, ncRNA metabolic process, ribosome biogenesis, ribonucleoprotein complex biogenesis, RNA binding, RNA methyltransferase activity and snoRNA binding, while the RBPs in module 2 were associated with translational elongation, mitochondrial translational elongation, cellular amide metabolic process, peptide biosynthetic process, structural molecule activity, and structural constituent of ribosome.

### Survival analysis of candidate hub RBPs

**Figure 3:**
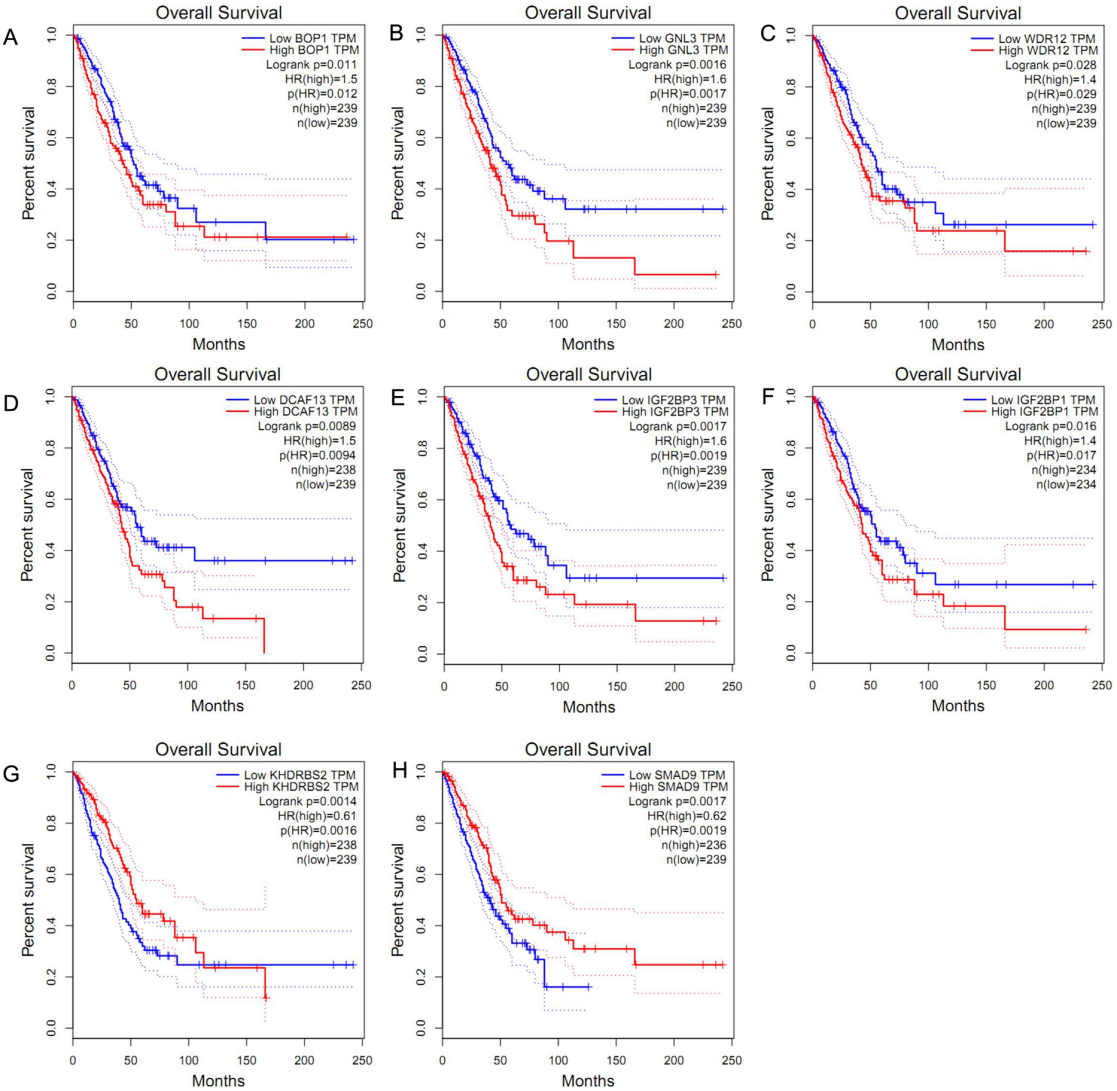
Survival analysis of hub RBPs. (A) *BOP1*, (B) *GNL3*, (C) *WDR12*, (D) *DCAF13*, (E) *IGF2BP3*, (F) *IGF2BP1*, (G) *KHDRBS2,* (H) *SMAD9*. The dotted lines represent the 95% confidence interval.

We obtained the hazard ratio (HR) of each candidate hub gene to OS by using GEPIA. The results showed that high expression of *BOP1* (HR = 1.5, *P* = 0.011), *GNL3* (HR = 1.6, *P* = 0.002), *WDR12* (HR = 1.4, *P* = 0.028), and *DCAF13* (HR = 1.5, *P* = 0.009) were significantly related to poor OS (Figs. 3A–3D). Considering that the candidate hub genes from PPI network were all up-regulated genes, we also executed survival analysis of the top ten over-expressed RBPs and top twenty down-expressed RBPs. We found that high expression of *IGF2BP3* (HR = 1.6, *P* = 0.002) or *IGF2BP1* (HR = 1.4, *P* = 0.016) was related to unfavorable prognosis of LUAD patients (Figs. 3E–3F), while the expression of *KHDRBS2* (HR = 0.61, *P* = 0.001) or *SMAD9* (HR = 0.62, *P* = 0.002) was negatively associated with OS (Figs. 3G–3H). These findings suggested that *BOP1, GNL3, WDR12, DCAF13, IGF2BP3, IGF2BP1, KHDRBS2* and *SMAD9* played a pivotal function in the development of lung adenocarcinoma.

### Hub RBPs verification and efficacy assessment

**Figure 4:**
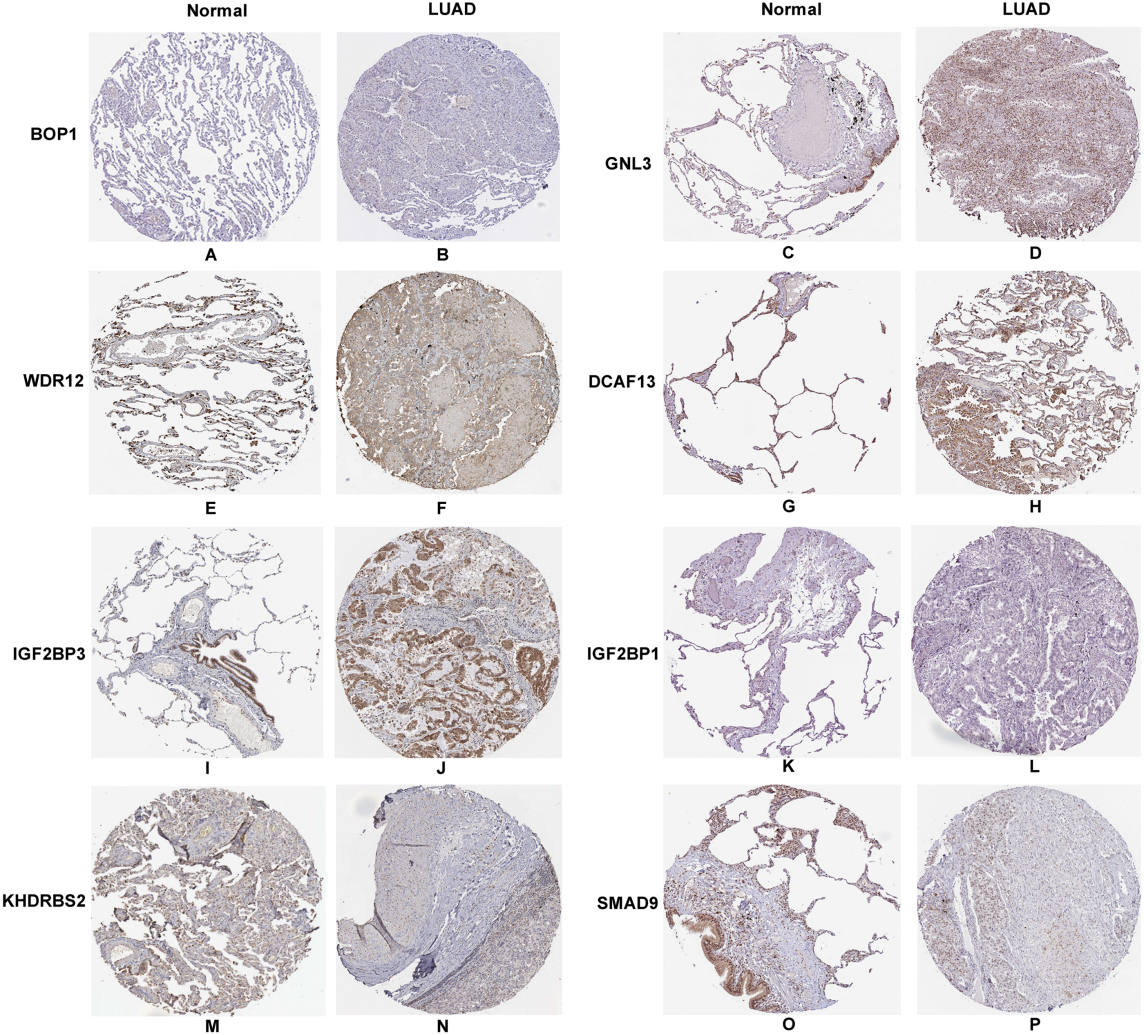
Validation the protein expression of hub genes in normal lung tissue and LUAD by the Human Protein Atlas database. (A–B) BOP1, (C–D) GNL3, (E–F) WDR12, (G–H) DCAF13, (I–J) IGF2BP3, (K–L) IGF2BP1, (M–N) KHDRBS2, (O–P) SMAD9.

**Figure 5:**
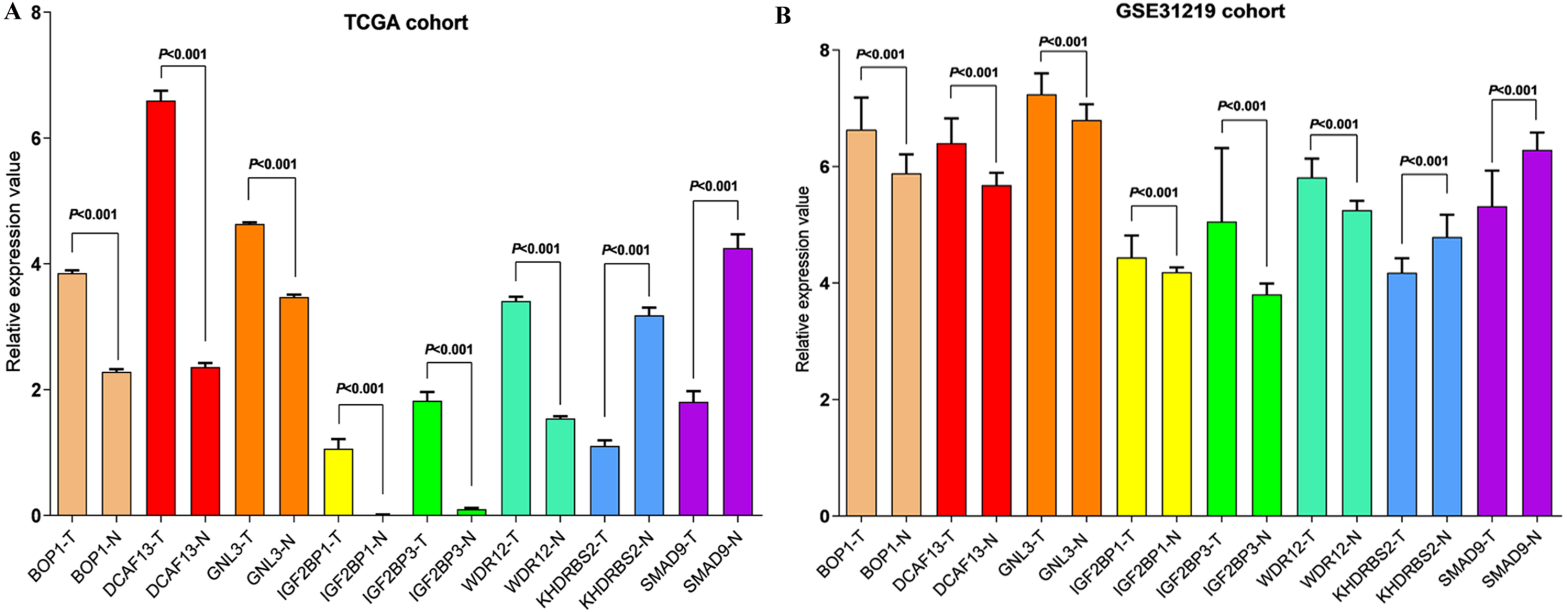
Validation the mRNA expression of hub RBPs in normal lung tissue and LUAD. (A) TCGA LUAD patients cohort; (B) GSE30219 LUAD patient cohort; T: tumor tissue; N: normal tissue.

**Figure 6:**
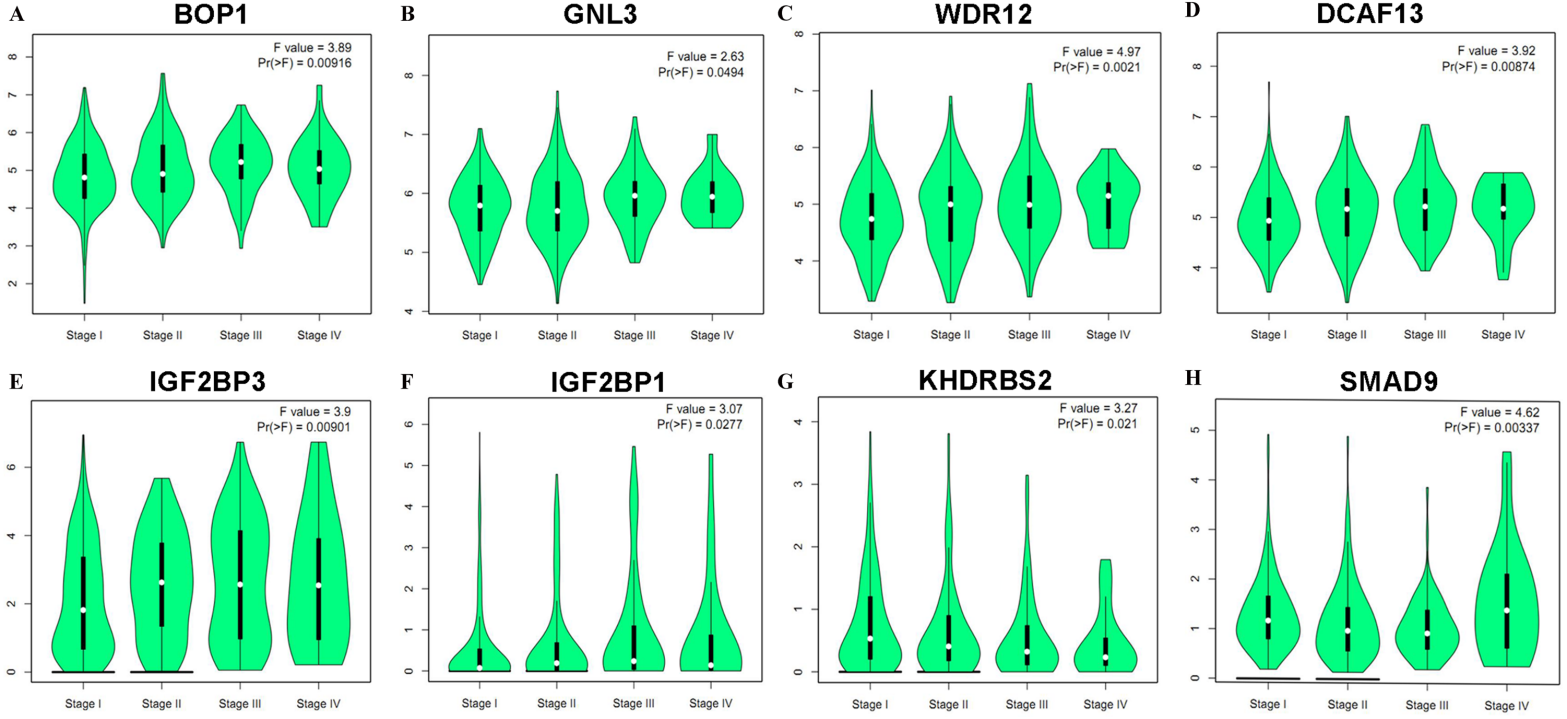
Correlation between eight hub RBPs expression and tumor stage in LUAD patients. (A) BOP1, (B) GNL3, (C) WDR12, (D) DCAF13, (E) IGF2BP3, (F) IGF2BP1, (G) KHDRBS2, (H) SMAD9.

**Figure 7:**
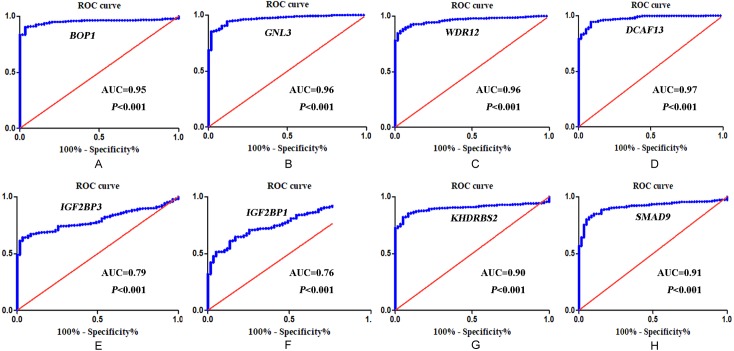
ROC analysis of eight hub RBPs based on TCGA database. (A) *BOP1*, (B) *GNL3*, (C) *WDR12*, (D) *DCAF13*, (E) *IGF2BP3*, (F) *IGF2BP1* ,** (G) *KHDRBS2,* (H) *SMAD9*.

**Figure 8:**
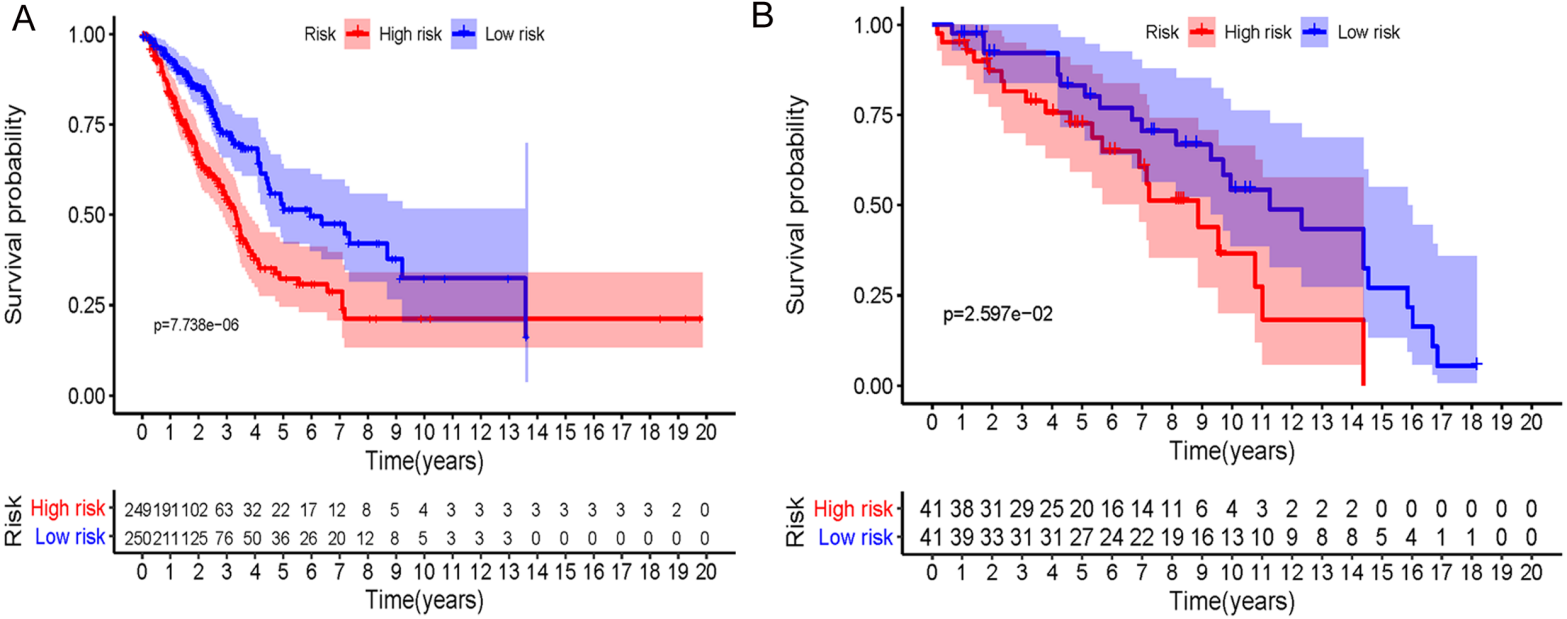
Prognostic risk assessment of integrated eight-hub genes for LUAD patients. (A) TCGA cohort, (B) GSE31219 cohort.

In accordance with the immunohistochemistry results from the Human Protein Atlas database, we found that the expression of hub RBPs BOP1, GNL3, WDR12, DCAF13, IGF2BP3, and IGF2BP1 were obviously elevated in lung carcinoma compared with normal lung tissues, while KHDRBS2 and SMAD9 were significantly decreased in tumor tissues (Fig. 4). Furthermore, we validated the expression level of the hub RBPs at transcription. The results indicated that the eight hub RBPs were deregulated in both TCGA and GSE30219 LUAD patient cohorts (Fig. 5). Moreover, the expression of the eight hub RBPs were significantly associated with tumor stage (Fig. 6). In addition, the ROC curve was used to assess the ability of eight hub RBPs to discriminate tumor tissue and normal lung tissue. The area under the curve (AUC) of hub genes *BOP1* (AUC = 0.95, 95%CI [0.9357–0.9700], *P*<0.0001), *GNL3* (AUC = 0.96, 95%CI [0.9509–0.9827], *P*<0.0001), *WDR12* (AUC = 0.9614, 95%CI [0.9459–0.9769], *P* <0.0001), *DCAF13* (AUC = 0.9747, 95%CI [0.9616–0.9879], *P* <0.0001), *IGF2BP3* (AUC = 0.7949, 95%CI [0.7565–0.8334], *P* <0.0001), *IGF2BP1* (AUC = 0.7754, 95%CI [0.7565–0.8334], *P* < 0.0001), *KHDRBS2* (AUC = 0.9018, 95%CI [0.8767–0.9270], *P* < 0.0001), and *SMAD9* (AUC = 0.9120, 95%CI [0.8855–0.9386], *P* < 0.0001) were greater than 0.7, suggesting that the eight hub genes with a better diagnostic accuracy for LUAD (Fig. 7). To further assess whether the eight hub genes can be used as one gene signature to predict the prognosis of LUAD patients, the risk score of every patient was calculated based on the coefficient value of the eight genes. Then, all patients were divided into high-risk and low-risk groups with the median risk score, and patients of high-risk were with poor OS compared with those of low-risk in both TCGA and GSE30219 cohorts (Fig. 8).

### Functions and pathways analysis of the alterations in candidate hub genes and their frequently changed neighbor genes in LUAD patients

**Figure 9:**
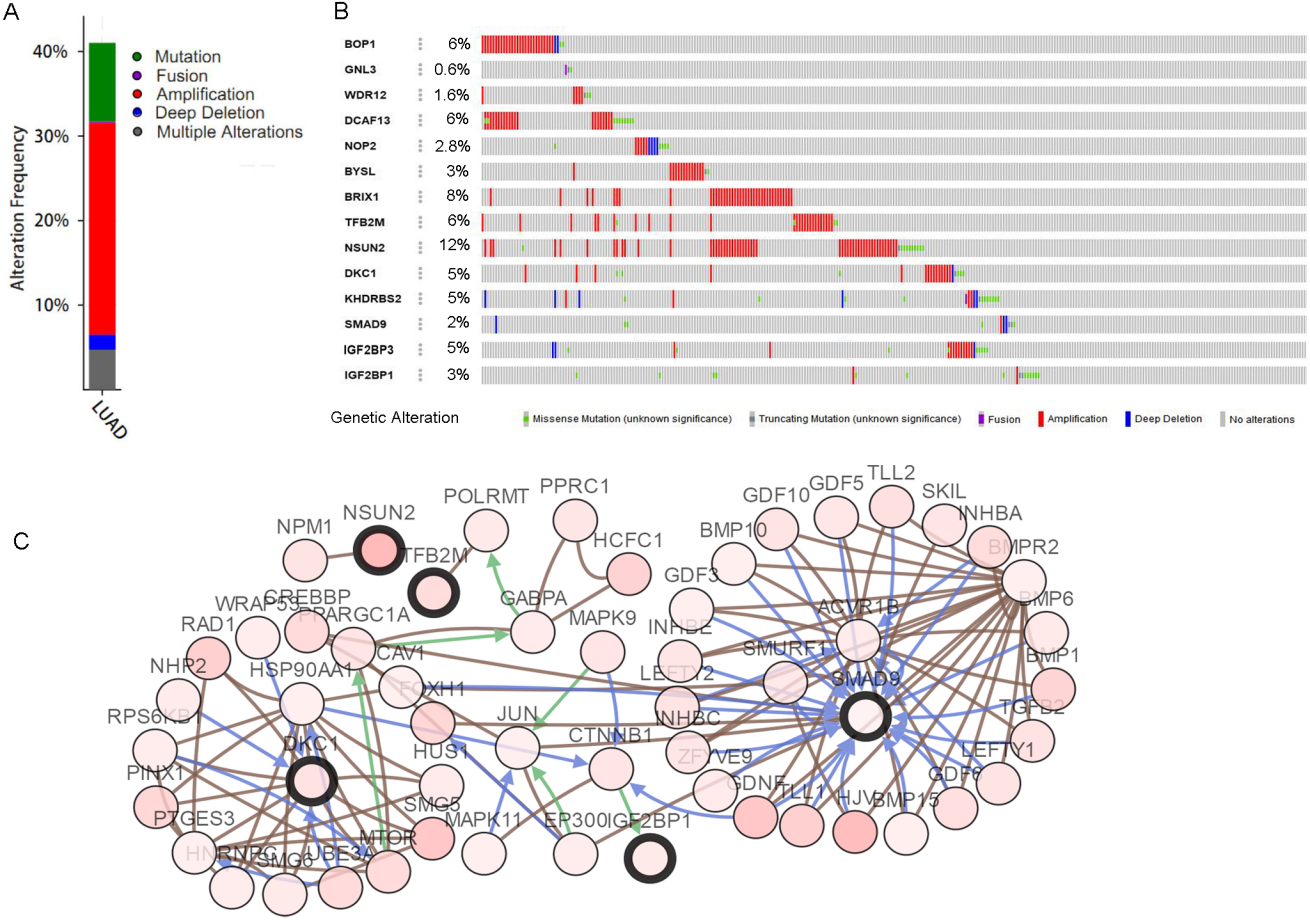
Hub RBPs expression and alteration a nalysis in lung adenocarcinoma. (A) Total alteration frequency; (B) genetic alteration of each hub gene; (C) co-expression network of hub RBPs and the 50 most frequently altered neighbor genes.

**Figure 10:**
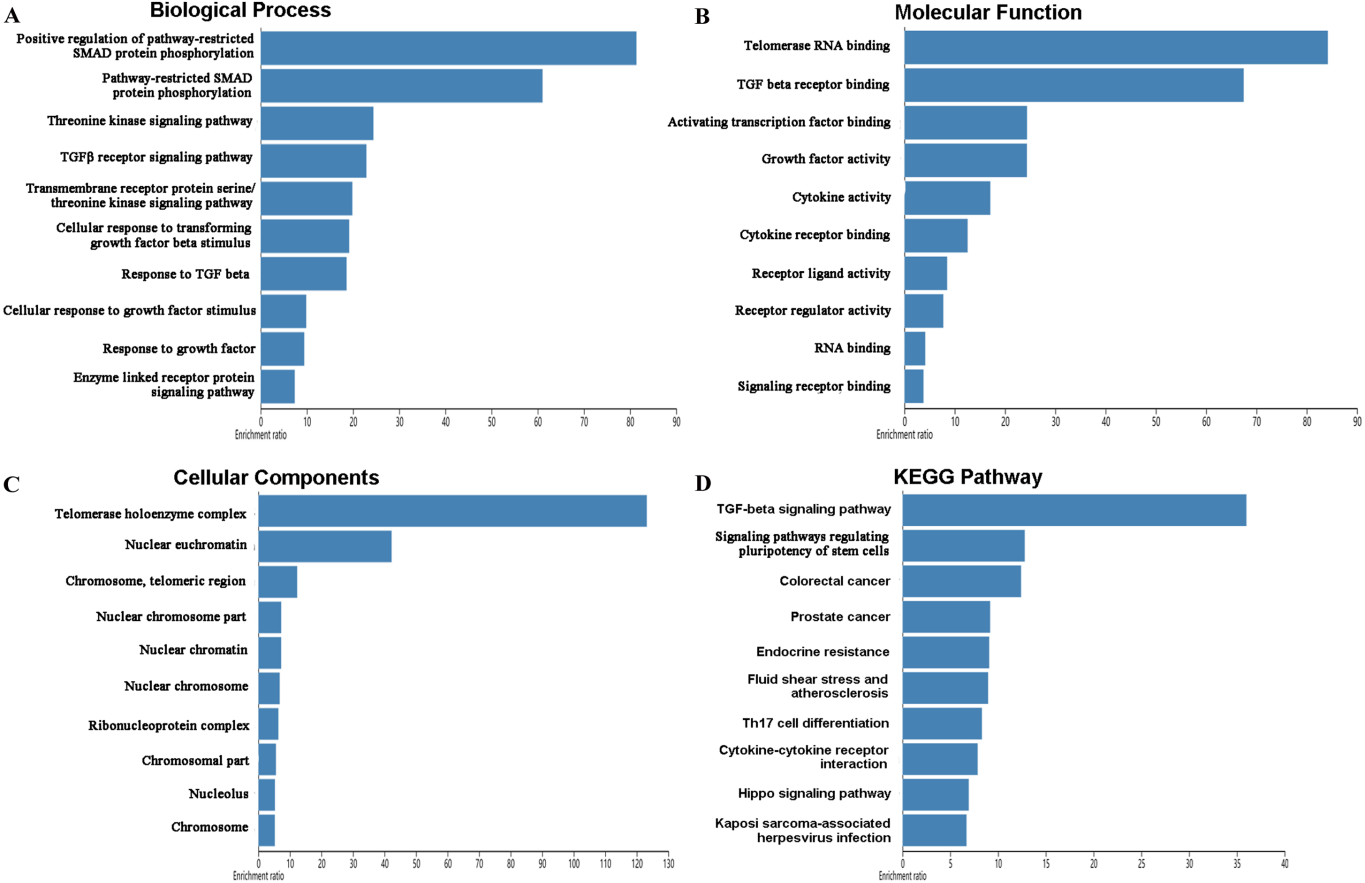
Functional enrichment analysis of key RBPs and genes significant associated with their alterations. (A) Biological process; (B) molecular function; (C) cellular components; (D) KEGG pathway.

The mutations and copy-number alterations analysis of the key genes *BOP1*, *GNL3*, *WDR12*, *NOP2*, *BYSL*, *BRIX1*, *DCAF13*, *TFB2M*, *NSUN2*, *DKC1*, *IGF2BP3*, *IGF2BP1*, *KHDRBS2* and *SMAD9* were carried out by using cBioPortal. We found that the 14 key genes altered in 223 samples out of 507 LUAD patients (Figs. 9A–9B). Then we constructed the interaction network contains 64 nodes, including 14 key genes and the 50 most frequently altered neighbor genes (Fig. 9C). The functions and pathways analysis of candidate hub genes and the genes significantly associated with hub genes alterations were conducted by using WebGestalt. The results showed that response to growth factor, enzyme linked receptor protein signaling pathway, TGF-beta signaling pathway, telomerase RNA binding, transmembrane receptor protein serine/threonine kinase signaling pathway, Th17 cell differentiation, and Hippo signaling pathway were significantly regulated by the hub genes alterations in LUAD (Fig. 10).

## Discussion

Despite advances in diagnostic and treatment methods over the past few decades, the overall mortality rates of lung carcinoma remained largely unchanged. Thence, understanding the etiology and molecular mechanism of lung carcinogenesis is necessary to improve the survival rate of patients. In recent years, many studies have shown that RBPs play a pivotal role in the development and progression of various human tumors (Pereira, Billaud & Almeida, 2017; Wu et al., 2019; Lujan, Ochoa & Hartley, 2018). However, the specific functional role of most RBPs in the process of tumorigenesis remains unclear (Chen et al., 2019). In this study, a total of 164 differently expressed RBPs between LUAD and normal lung tissue were screened, consisting of 124 up-regulated RBPs and 40 down-regulated RBPs. Then, we systematically explored the potential functional pathways and constructed a PPI network of these differently expressed RBPs. Moreover, the module analysis, survival analysis, ROC analysis and copy-number alterations analysis of hub RBPs were performed to further explore their biological functions and clinical significance. These findings may contribute to develop novel biomarkers for the diagnosis and prognosis of LUAD patients.

The function enrichment analysis demonstrated that the differently expressed RBPs were significantly related to RNA processing, RNA metabolic process, RNA binding, peptide biosynthetic process, regulation of translation, regulation of cellular amide metabolic process, catalytic activity acting on RNA, single-stranded RNA binding, poly-pyrimidine tract binding, RNA cap binding complex, ribonucleoprotein complex, mRNA 3′-UTR binding, ribonucleoprotein granule, and nucleolus. Previous studies have shown that RNA metabolism and RNA processing have been increasingly recognized in various diseases (Martinez-Terroba et al., 2018; Hinkle et al., 2019; Jain et al., 2019). RBPs can bind to mRNAs to form ribonucleoprotein complexes and regulate their expression by increasing mRNA stability, which play important roles in the development of many diseases. A recent study has revealed that RNA-binding protein SRSF1 promote tumor cell proliferation and progression by increasing LIG1 mRNA stability in non-small cell lung cancer (Martinez-Terroba et al., 2018). RNA binding proteins in the nucleus play key effects in regulated mRNA alternative splicing process and lead to alter in the expression of tumor-associated genes (Shao et al., 2019). Additionally, ribonucleoprotein granule is an important area that implements protein synthesis. The mutation of ribonucleoprotein regulates the translation process and associated with tumor development (Goudarzi & Lindstrom, 2016). It has been reported that heterogeneous nuclear ribonucleoprotein A2B1 is over-expressed in tissues and blood of patients with lung cancer, and which contributes to lung tumorigenesis (Dowling et al., 2015). The KEGG pathways analysis suggested that the differently expressed RBPs regulate the occurrence and development of lung cancer by affecting RNA degradation, TGF-beta signaling pathway, RNA transport, aminoacyl-tRNA biosynthesis, and mRNA surveillance pathway.

Furthermore, we constructed a PPI network of these differently expressed RBPs and got the top ten hub genes including *BOP1*, *GNL3*, *WDR12*, *NOP2*, *BYSL*, *BRIX1*, *DCAF13*, *TFB2M*, *NSUN2* and *DKC1*. Among these genes, *NOP2*, also known as p120, is found highly expressed in lung adenocarcinoma tissue and negatively associated with patients prognosis (Saijo et al., 2001; Uchiyama et al., 1997). Despite the correlation between most RBPs and LUAD still unclear, several RBPs have been reported to be closely associated with other cancers. *BOP1* as Wnt/*β*-catenin target gene involved in induced migration, EMT, and metastasis of colorectal carcinoma (Qi et al., 2016). *GNL3* is upregulated in colon carcinoma cell and tissue, and facilitate tumor cell epithelial-mesenchymal transition by activating the Wnt/*β*-catenin signaling pathway (Tang et al., 2017a). *WDR12* as a new oncogene contributes to liver cancer spread (Yin et al., 2018). *DCAF13* is upregulated in liver cancer and significantly related to poor survival (Cao et al., 2017; Qiao et al., 2019). NSUN2 as a tRNA methyltransferase, is significantly related to cancer progression in several tumors, such as esophageal squamous cell carcinoma (Li et al., 2018), breast cancer (Yi et al., 2017), and ovarian cancer (Yang et al., 2017). *DKC1* is deregulated in glioma and knockdown of *DKC1* restrain cancer cell proliferation, invasion and migration (Miao et al., 2019). By executing module analysis of PPI network, we found that LUAD is associated with RNA metabolic process, ribosome biogenesis, cellular amide metabolic process, peptide biosynthetic process, and translational elongation.

In addition, the survival analysis showed that 8 RBPs are obviously related to survival of LUAD patients. Increased expression of *BOP1, GNL3, WDR12, DCAF13, IGF2BP3* and *IGF2BP1* were related to poor overall survival, while overexpression of KHDRBS2 and SMAD were related to better overall survival. Subsequently, we verified the expression patterns of these eight hub RBPs on translation and transcription level by using the Human Protein Atlas database and GEPIA database, the results showed that BOP1, GNL3, WDR12, DCAF13, IGF2BP3 and IGF2BP1 were upregulated, and KHDRBS2 as well as SMAD were downregulated in LUAD tissues. These findings suggested that *BOP1, GNL3, WDR12, DCAF13, IGF2BP3* and *IGF2BP1* may have potential carcinogenic effects, *KHDRBS2* and *SMAD* role as tumor suppressor genes. Previous study has reported that the expression IGF2BP3 or IGF2BP1 can predict poor overall survival in lung cancer patients (Shi et al., 2017), which were consistent with our results. Two other studies also showed that *DCAF13* was increased in hepatocellular carcinoma or breast cancer, and obviously associated with poor overall survival (Cao et al., 2017; Wang et al., 2019). The ROC curve analysis revealed that these 8 RBPs with better diagnostic accuracy for distinguishing LUAD patients from healthy people, which could be used as potential diagnostic molecular markers in the future. Besides, we found that the eight hub genes can be used as one gene signature to predict the prognosis of LUAD patients in TCGA and GSE30219 cohorts.

Finally, we analyzed the hub genes alterations and constructed the co-expression network by using the cBioPortal online tool for LUAD. The results showed that amplification of *NSUN2* was the most common alterations in all copy number variations of these RBPs. A study revealed that *NSUN2* gene copy number was elevated in colorectal and oral carcinoma (Okamoto et al., 2012).We also found that *DCAF13* and *BOP1* have high copy number gain frequency in patients with LUAD. Previous studies have shown that *DCAF13* is amplified in various tumors, such as hepatocellular carcinoma (Cao et al., 2017), breast cancer (Wang et al., 2019), and osteosarcoma (Chen et al., 2018). Another research has demonstrated that dosage increase of the *BOP1* was a frequent event in colorectal carcinoma and related to cancer occurrence (Killian et al., 2006). By constructing a protein co-expression network of key 14 RBPs and the 50 most frequently altered neighbor genes, we found that the alterations of hub RBPs in LUAD were involved in regulating enzyme linked receptor protein signaling pathway, telomerase RNA binding, TGF-beta signaling pathway, Hippo signaling pathway, and transmembrane receptor protein serine/threonine kinase signaling pathway. These results indicated that mutations and copy-number alterations of RBPs were play important roles in lung carcinogenesis and progression.

In conclusion, the current study conducted a comprehensive bioinformatics analysis of differently expressed RBPs to identify the potential biomarkers and predict progression of LUAD. The results showed that the expression of *BOP1, GNL3, WDR12, DCAF13, IGF2BP3, IGF2BP1, KHDRBS2* and *SMAD* were significantly related to prognosis of LUAD patient. These findings have the potential to provide new therapeutic targets prognostic markers for LUAD.

##  Supplemental Information

10.7717/peerj.8509/supp-1 Table S1 The differentially expressed RBPs in LUAD

## Additional Information and Declarations

### Data Availability

The following information was supplied regarding data availability: The raw data can be found at TCGA (https://portal.gdc.cancer.gov/projects/TCGA-LUAD) and GEO: GSE30219.
