# Supplementary material for: Integrated analysis of the roles and prognostic value of RNA binding proteins in lung adenocarcinoma
Source: PeerJ. 2020 Feb 6;8:e8509. doi: 10.7717/peerj.8509 (PMC7007976; doi:10.7717/peerj.8509)
Supplement: Table S1 [file peerj-08-8509-s001.docx]

Table S1 The differentially expressed RBPs in LUAD

| Gene name | log2FC | *P*-value | Adj. *P* | Alteration | Gene name | log2FC | *P*-value | Adj. *P* | Alteration |
| --- | --- | --- | --- | --- | --- | --- | --- | --- | --- |
| SMAD6 | -2.56 | 1.67E-90 | 5.20E-88 | Down | LIN28B | 7.99 | 6.51E-23 | 6.61E-22 | Up |
| NOVA2 | -2.41 | 7.52E-82 | 1.40E-79 | Down | APOBEC1 | 6.79 | 8.82E-34 | 1.74E-32 | Up |
| AFF3 | -2.38 | 1.28E-28 | 1.86E-27 | Down | IGF2BP1 | 6.60 | 6.92E-54 | 3.79E-52 | Up |
| PIH1D3 | -2.24 | 9.07E-11 | 3.63E-10 | Down | EEF1A2 | 6.38 | 3.49E-84 | 7.14E-82 | Up |
| PTRF | -2.08 | 2.67E-68 | 2.77E-66 | Down | NR0B1 | 6.10 | 4.03E-21 | 3.60E-20 | Up |
| TRIM71 | -2.00 | 1.27E-11 | 5.52E-11 | Down | TERT | 5.35 | 1.14E-59 | 7.89E-58 | Up |
| NXF3 | -1.94 | 4.35E-12 | 1.96E-11 | Down | PIWIL3 | 5.34 | 1.94E-15 | 1.15E-14 | Up |
| L1TD1 | -1.88 | 1.17E-21 | 1.09E-20 | Down | PIWIL1 | 5.10 | 4.90E-27 | 6.49E-26 | Up |
| APOBEC4 | -1.84 | 9.02E-08 | 2.69E-07 | Down | EIF4E1B | 4.83 | 1.76E-13 | 8.95E-13 | Up |
| TLR8 | -1.82 | 1.74E-26 | 2.23E-25 | Down | YBX2 | 4.75 | 1.77E-65 | 1.62E-63 | Up |
| ZFP36 | -1.81 | 7.35E-30 | 1.16E-28 | Down | DDX53 | 4.62 | 8.54E-12 | 3.76E-11 | Up |
| ADARB1 | -1.76 | 7.04E-65 | 6.20E-63 | Down | KHDC1L | 4.50 | 3.48E-20 | 2.89E-19 | Up |
| KHDRBS2 | -1.75 | 1.41E-12 | 6.64E-12 | Down | LIN28A | 4.34 | 1.86E-14 | 1.02E-13 | Up |
| ENDOU | -1.59 | 5.01E-20 | 4.13E-19 | Down | MEX3A | 4.19 | 7.01E-112 | 9.14E-109 | Up |
| NCBP2L | -1.53 | 1.04E-09 | 3.80E-09 | Down | IGF2BP3 | 4.00 | 2.97E-48 | 1.26E-46 | Up |
| RBMS3 | -1.53 | 2.62E-24 | 2.93E-23 | Down | TDRD5 | 3.67 | 1.39E-32 | 2.57E-31 | Up |
| ZCCHC5 | -1.48 | 6.58E-13 | 3.19E-12 | Down | A1CF | 3.61 | 2.02E-17 | 1.38E-16 | Up |
| PPARGC1B | -1.42 | 3.23E-26 | 4.05E-25 | Down | RDM1 | 3.60 | 1.84E-66 | 1.74E-64 | Up |
| SMAD9 | -1.42 | 1.49E-15 | 8.94E-15 | Down | EXO1 | 3.59 | 3.12E-98 | 1.43E-95 | Up |
| SECISBP2L | -1.38 | 2.36E-27 | 3.19E-26 | Down | CELF3 | 3.49 | 1.22E-19 | 9.77E-19 | Up |
| QKI | -1.35 | 8.92E-50 | 4.06E-48 | Down | ELAVL2 | 3.45 | 1.66E-24 | 1.88E-23 | Up |
| ADARB2 | -1.30 | 1.47E-08 | 4.77E-08 | Down | CELF5 | 3.36 | 2.10E-38 | 5.31E-37 | Up |
| ZNF106 | -1.30 | 5.57E-48 | 2.32E-46 | Down | RBFOX1 | 3.30 | 4.36E-10 | 1.64E-09 | Up |
| RBMS2 | -1.24 | 5.91E-49 | 2.59E-47 | Down | ERN2 | 3.06 | 3.23E-20 | 2.70E-19 | Up |
| CTIF | -1.21 | 5.59E-43 | 1.80E-41 | Down | DQX1 | 3.04 | 3.05E-27 | 4.08E-26 | Up |
| SAMHD1 | -1.21 | 5.75E-31 | 9.71E-30 | Down | BOLL | 2.81 | 1.26E-11 | 5.47E-11 | Up |
| CELF2 | -1.20 | 2.43E-21 | 2.20E-20 | Down | DDX4 | 2.73 | 4.10E-15 | 2.37E-14 | Up |
| SMAD7 | -1.11 | 3.76E-38 | 9.35E-37 | Down | EZH2 | 2.55 | 6.57E-91 | 2.07E-88 | Up |
| EIF4E3 | -1.11 | 3.00E-29 | 4.56E-28 | Down | RNASE10 | 2.54 | 6.26E-13 | 3.04E-12 | Up |
| CPEB1 | -1.10 | 1.87E-08 | 5.99E-08 | Down | RNF113B | 2.52 | 9.34E-07 | 2.48E-06 | Up |
| SIDT2 | -1.09 | 7.00E-46 | 2.65E-44 | Down | MKRN3 | 2.50 | 5.26E-12 | 2.36E-11 | Up |
| RBM24 | -1.09 | 6.65E-06 | 1.59E-05 | Down | TDRD1 | 2.49 | 5.66E-15 | 3.24E-14 | Up |
| ZC3H12B | -1.08 | 2.97E-15 | 1.74E-14 | Down | MSI1 | 2.33 | 2.15E-16 | 1.36E-15 | Up |
| IFIT1B | -1.06 | 0.00316 | 0.0052 | Down | PABPC1L2B | 2.32 | 0.012499 | 0.018567 | Up |
| OASL | -1.05 | 1.77E-09 | 6.29E-09 | Down | RPL39L | 2.31 | 1.19E-38 | 3.06E-37 | Up |
| RNASE13 | -1.05 | 0.00021 | 0.00042 | Down | SRRM3 | 2.30 | 6.00E-40 | 1.65E-38 | Up |
| NXF5 | -1.03 | 0.00038 | 0.00071 | Down | RBM46 | 2.26 | 7.67E-08 | 2.30E-07 | Up |
| ZCCHC24 | -1.02 | 8.29E-29 | 1.22E-27 | Down | PABPC1L | 2.15 | 7.79E-42 | 2.36E-40 | Up |
| PTRH1 | -1.02 | 9.32E-13 | 4.46E-12 | Down | SRSF12 | 2.12 | 1.52E-29 | 2.36E-28 | Up |
| SRRM4 | -1.01 | 0.00435 | 0.00698 | Down | CALR3 | 2.10 | 6.58E-09 | 2.21E-08 | Up |
| DNMT3B | 2.06 | 3.39E-35 | 7.31E-34 | Up | EXOSC5 | 1.18 | 2.09E-26 | 2.66E-25 | Up |
| MAEL | 1.98 | 1.36E-10 | 5.37E-10 | Up | TFB2M | 1.18 | 3.76E-45 | 1.36E-43 | Up |
| Gene name | log2FC | *P*-value | Adj.*P* | Alteration | Gene name | log2FC | *P*-value | Adj.*P* | Alteration |
| PABPC1L2A | 1.96 | 0.02252 | 0.03204 | Up | CD3EAP | 1.17 | 1.24E-31 | 2.17E-30 | Up |
| RPL3L | 1.90 | 5.57E-19 | 4.27E-18 | Up | VARS | 1.17 | 2.58E-40 | 7.19E-39 | Up |
| ZFR2 | 1.89 | 8.27E-08 | 2.48E-07 | Up | POLR2H | 1.17 | 2.13E-46 | 8.27E-45 | Up |
| ELAVL4 | 1.75 | 1.38E-14 | 7.61E-14 | Up | RPL22L1 | 1.17 | 5.03E-18 | 3.60E-17 | Up |
| GAPDH | 1.71 | 3.00E-45 | 1.09E-43 | Up | INTS8 | 1.16 | 5.62E-47 | 2.24E-45 | Up |
| BOP1 | 1.71 | 1.50E-44 | 5.29E-43 | Up | BARD1 | 1.14 | 1.06E-30 | 1.76E-29 | Up |
| DAZL | 1.70 | 5.59E-07 | 1.52E-06 | Up | SNRPE | 1.13 | 7.04E-39 | 1.82E-37 | Up |
| NPM3 | 1.69 | 3.65E-46 | 1.41E-44 | Up | DKC1 | 1.13 | 1.20E-47 | 4.98E-46 | Up |
| ZNF239 | 1.67 | 1.66E-47 | 6.83E-46 | Up | CNOT11 | 1.12 | 7.24E-68 | 7.37E-66 | Up |
| ZC3HAV1L | 1.57 | 5.57E-45 | 2.00E-43 | Up | URB1 | 1.12 | 3.06E-30 | 4.96E-29 | Up |
| TDRD15 | 1.56 | 0.00188 | 0.00312 | Up | MRPL15 | 1.12 | 7.41E-26 | 9.07E-25 | Up |
| RPP40 | 1.53 | 6.52E-46 | 2.47E-44 | Up | ESRP1 | 1.12 | 6.76E-35 | 1.43E-33 | Up |
| BRCA1 | 1.46 | 3.66E-29 | 5.53E-28 | Up | MEX3B | 1.11 | 2.26E-21 | 2.05E-20 | Up |
| AZGP1 | 1.43 | 1.29E-05 | 2.97E-05 | Up | DUS4L | 1.10 | 4.02E-36 | 9.08E-35 | Up |
| NANOS1 | 1.40 | 4.43E-22 | 4.24E-21 | Up | PABPC3 | 1.10 | 6.36E-11 | 2.58E-10 | Up |
| TDRD12 | 1.38 | 1.25E-07 | 3.66E-07 | Up | KHDC1 | 1.09 | 2.68E-21 | 2.42E-20 | Up |
| TDRKH | 1.37 | 3.85E-46 | 1.48E-44 | Up | GNL3 | 1.09 | 1.34E-45 | 4.96E-44 | Up |
| METTL1 | 1.37 | 1.34E-33 | 2.62E-32 | Up | PRKDC | 1.09 | 8.03E-25 | 9.29E-24 | Up |
| IPO4 | 1.36 | 1.97E-41 | 5.86E-40 | Up | NOP2 | 1.09 | 6.86E-30 | 1.09E-28 | Up |
| DCAF13 | 1.33 | 2.48E-47 | 1.00E-45 | Up | MRPL24 | 1.09 | 6.04E-26 | 7.44E-25 | Up |
| RPL10L | 1.33 | 0.00052 | 0.00096 | Up | JAKMIP1 | 1.09 | 2.12E-09 | 7.47E-09 | Up |
| RNASE7 | 1.30 | 0.00024 | 0.00047 | Up | TBRG4 | 1.08 | 1.06E-37 | 2.56E-36 | Up |
| BZW2 | 1.30 | 1.06E-45 | 3.97E-44 | Up | CSTF2 | 1.08 | 9.47E-52 | 4.76E-50 | Up |
| FBLL1 | 1.30 | 5.64E-05 | 0.00012 | Up | HEATR1 | 1.07 | 9.06E-40 | 2.46E-38 | Up |
| RNASEH2A | 1.29 | 4.72E-29 | 7.08E-28 | Up | MTG1 | 1.06 | 1.02E-25 | 1.24E-24 | Up |
| MRM1 | 1.27 | 3.42E-44 | 1.18E-42 | Up | MRPL36 | 1.05 | 3.12E-22 | 3.02E-21 | Up |
| PUS7 | 1.26 | 5.57E-43 | 1.80E-41 | Up | ILF2 | 1.05 | 3.73E-36 | 8.46E-35 | Up |
| NOVA1 | 1.23 | 7.07E-07 | 1.90E-06 | Up | GARS | 1.05 | 2.79E-34 | 5.69E-33 | Up |
| MRPL12 | 1.22 | 1.02E-22 | 1.02E-21 | Up | EXOSC4 | 1.05 | 3.24E-20 | 2.70E-19 | Up |
| BRIX1 | 1.22 | 7.22E-37 | 1.69E-35 | Up | PABPC1 | 1.05 | 2.01E-27 | 2.73E-26 | Up |
| BYSL | 1.22 | 4.99E-33 | 9.50E-32 | Up | AIMP2 | 1.05 | 1.13E-30 | 1.87E-29 | Up |
| DARS2 | 1.21 | 1.44E-38 | 3.65E-37 | Up | TARS2 | 1.04 | 9.58E-44 | 3.24E-42 | Up |
| MOV10L1 | 1.21 | 5.94E-09 | 2.00E-08 | Up | MATR3 | 1.04 | 3.72E-18 | 2.68E-17 | Up |
| SPATS2 | 1.21 | 7.99E-47 | 3.17E-45 | Up | AC004381.6 | 1.02 | 1.34E-22 | 1.33E-21 | Up |
| DUS1L | 1.20 | 8.98E-41 | 2.56E-39 | Up | XPO5 | 1.02 | 5.00E-38 | 1.23E-36 | Up |
| PUSL1 | 1.19 | 3.32E-30 | 5.36E-29 | Up | MRPL3 | 1.02 | 2.13E-42 | 6.63E-41 | Up |
| MSI2 | 1.19 | 2.41E-40 | 6.71E-39 | Up | NSUN2 | 1.02 | 3.25E-33 | 6.26E-32 | Up |
| C2orf15 | 1.19 | 1.72E-27 | 2.35E-26 | Up | CLK2 | 1.02 | 2.47E-37 | 5.89E-36 | Up |
| OAS1 | 1.18 | 4.21E-15 | 2.43E-14 | Up | WDR12 | 1.00 | 1.36E-37 | 3.28E-36 | Up |
| PUS1 | 1.18 | 4.96E-35 | 1.06E-33 | Up | CPSF4L | 1.00 | 0.00023 | 0.00045 | Up |
